# Analysis of US County Characteristics and Clinicians With Waivers to Prescribe Buprenorphine After Changes in Federal Education Requirements

**DOI:** 10.1001/jamanetworkopen.2022.37912

**Published:** 2022-10-21

**Authors:** Thuy Nguyen, Barbara Andraka-Christou, Camila Arnaudo, W. David Bradford, Kosali Simon, Joanne Spetz

**Affiliations:** 1 Department of Health Management and Policy, School of Public Health, University of Michigan, Ann Arbor; 2 School of Global Health Management and Informatics, University of Central Florida, Orlando; 3 Indiana University Health, Bloomington; 4 Department of Public Administration and Policy, University of Georgia, Athens; 5 O’Neil School of Public and Environmental Affairs, Indiana University, Bloomington; 6 National Bureau of Economic Research, Cambridge, Massachusetts; 7 Philip R. Lee Institute for Health Policy Studies, University of California, San Francisco

## Abstract

This cross-sectional study investigates the growth in the number of clinicians in the US who obtained waivers for prescribing buprenorphine after the elimination of federal educational requirements.

## Introduction

Buprenorphine is associated with decreased mortality from opioid use disorder,^1^ but prescribing is limited in office-based settings to clinicians with federal waivers. To expand this workforce, on April 28, 2021, the US federal government eliminated educational requirements for waivers to prescribe buprenorphine to 30 or fewer patients.^2^ Modest growth in the nationwide number of clinicians with waivers was observed after this exemption^3^; however, geographic variation in the net growth of this workforce is unknown. Characterization of counties with recent net growth in clinicians with waivers may help address geographically varying shortages of buprenorphine prescribers and opioid overdose deaths.^4^

## Methods

Using the Controlled Substances Act Registration Information Database and stratifying by rurality and types of clinicians, we compared 12-month net growth in waivers among clinicians after the exemption (ie, raw changes between March 31, 2021, [2021 quarter 1 (Q1)] and March 31, 2022 [2022 Q1]) in rural vs urban counties. We conducted multivariate linear regressions of this outcome measure on quartiles of the key factors: preexisting rate of clinicians with waivers (ie, ranking counties by the number of clinicians with waivers per capita in 2021 Q1), physician-to-population ratios, and shares of racial and ethnic minority group population (eMethods 1-3 in the Supplement). Racial and ethnic data were obtained from the County Health Rankings Database as important demographic information to characterize counties with more or less expanded workforce of clinicians with waivers. We used Stata/MP, version 17.0 (StataCorp LLC) to run analyses and 2-sided hypothesis tests (α = .05). The University of Michigan Institutional Review Board exempted analyses from review and informed consent because the data set is publicly available and data are deidentified. We followed the STROBE reporting guideline.

## Results

While the nationwide number of clinicians with waivers grew from 45 790 in 2018 Q1 to 114 493 in 2022 Q1, the mean quarterly growth rates declined (8.3% per quarter from 2018 Q1 to 2021 Q1 and 4.1% per quarter from 2021 Q2 to 2022 Q1) (Figure). The net growth of waivers varied by rurality and the preexisting waiver rate. Between 2021 Q1 and 2022 Q1, mean waiver net growth in rural counties was considerably lower and driven by increases in advanced practice nurses (APNs) and physicians’ assistants (PAs) compared with urban counties. A mean of 5.0 clinicians with waivers per 100 000 population (1.3 physicians and 3.7 APNs and PAs) were added in the top quartile of preexisting waiver rates for rural counties (vs 0.12 clinicians in the lowest quartile). By contrast, 8.4 clinicians per capita (4.2 physicians and 4.2 APNs and PAs) were added in the top quartile of urban counties (vs 0.7 clinicians in the lowest quartile).

**Figure.**
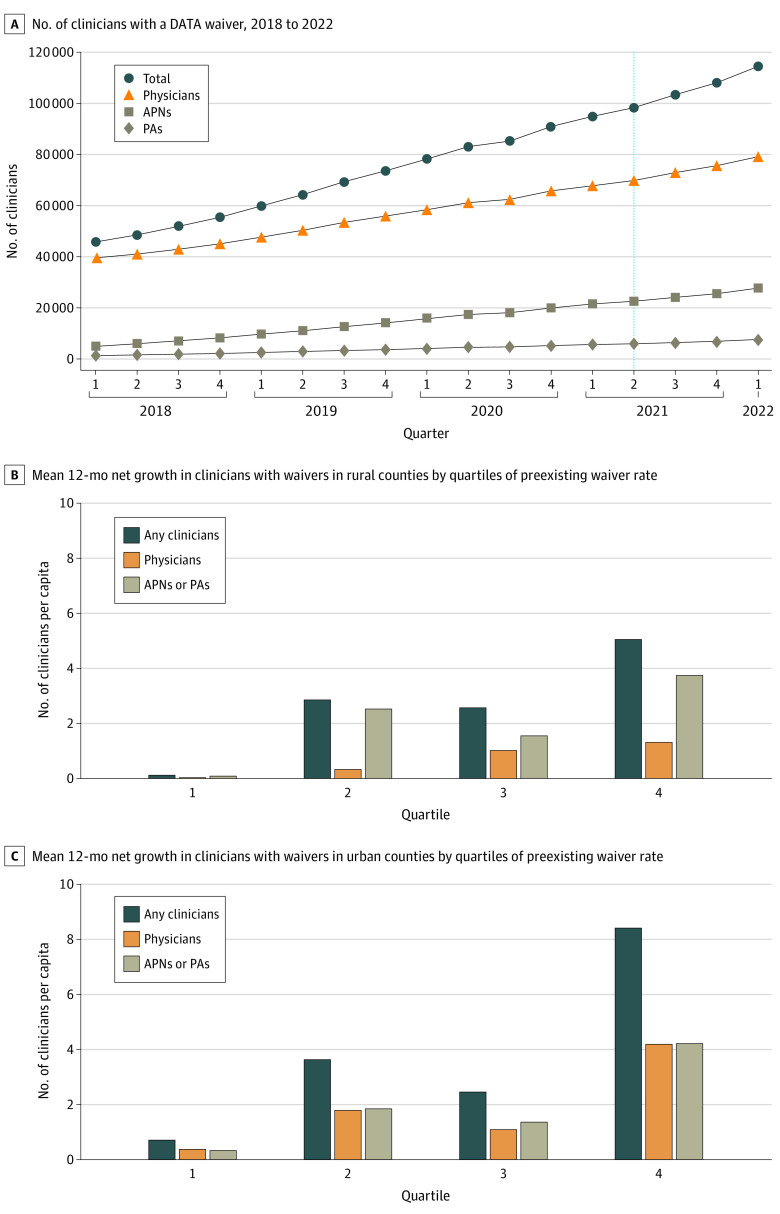
Changes in National- and County-Level Buprenorphine Waiver Capacity in the US by Clinician Type After the 2021 Federal Training Exemption A, Number of clinicians who were granted a waiver to prescribe buprenorphine for opioid use disorder. The vertical line delineates the timing of the 2021 training exemption (April 28, 2021). B and C, Unadjusted mean 12-month net growth from March 2021 to March 2022 in county-level buprenorphine treatment capacity among rural (B) and urban (C) counties. The per capita measure is the number of clinicians per 100 000 residents. APN indicates advanced practice nurse (including all advanced practice nurses, midwives, clinical nurse specialists, and nurse anesthetists); DATA, Drug Addiction Treatment Act; and PA, physician assistant.

Regression analyses indicated an association between preexisting waiver rate and net growth of clinicians with waivers in urban counties (5.5 [95% CI, 4.3-6.8] additional clinicians in the top vs lowest quartile) and rural counties (3.4 [95% CI, 1.3-5.5] clinicians), owing largely to 2.9 (95% CI, 1.4-4.4) additional APNs and PAs. Compared with the lowest quartile, rural counties with the highest physician-to-population ratios netted 5.4 (95% CI, 2.8-8.0) clinicians and 3.8 (95% CI, 1.6-5.9) physicians, while urban counties netted 2.3 (95% CI, 1.0-3.5) clinicians. The share of racial and ethnic minority group population was associated with net growth in urban counties (1.5 [95% CI, 0.2-2.9] clinicians in the top vs lowest quartile) but was not associated with rural growth (Table).

**Table.** Associations Between County Factors and Clinician Type^a^

Factor	Clinician type					
	Among 1802 metropolitan and micropolitan counties			Among 1328 rural counties		
	Any clinician	Physician	APN and PA	Any clinician	Physician	APN and PA
Preexisting rate of clinicians with waivers by quartile						
Quartile 1 (highest)	5.5 (4.3 to 6.8)	2.6 (1.8 to 3.4)	2.9 (2.0 to 3.8)	3.4 (1.3 to 5.5)	0.6 (−1.0 to 2.1)	2.9 (1.4 to 4.4)
Quartile 2	1.4 (0.8 to 1.9)	0.5 (0.07 to 0.9)	0.9 (0.4 to 1.4)	1.7 (0.3 to 3.1)	−0.2 (−1.0 to 0.5)	1.9 (0.7 to 3.2)
Quartile 3	1.0 (0.4 to 1.6)	0.2 (−0.1 to 0.6)	0.7 (0.4 to 1.1)	1.6 (0.7 to 2.5)	0.5 (−0.1 to 1.2)	1.1 (0.5 to 1.6)
Quartile 4 (lowest)	1 [Reference]	1 [Reference]	1 [Reference]	1 [Reference]	1 [Reference]	1 [Reference]
Physicians per 100 000 population by quartile						
Quartile 1 (highest)	2.3 (1.0 to 3.5)	1.1 (0.3 to 1.8)	1.2 (0.3 to 2.1)	5.4 (2.8 to 8.0)	3.8 (1.6 to 5.9)	1.6 (−0.1 to 3.3)
Quartile 2	0.9 (0.1 to 1.7)	0.2 (−0.3 to 0.7)	0.7 (0.1 to 1.3)	1.8 (0.6 to 3.0)	1.4 (0.5 to 2.2)	0.5 (−0.6 to 1.5)
Quartile 3	0.7 (−0.002 to 1.4)	0.08 (−0.3 to 0.5)	0.6 (0.09 to 1.1)	0.4 (−0.7 to 1.4)	0.5 (−0.05 to 1.0)	−0.07 (−1.2 to 1.0)
Quartile 4 (lowest)	1 [Reference]	1 [Reference]	1 [Reference]	1 [Reference]	1 [Reference]	1 [Reference]
Percent of racial or ethnic minority group population by quartile						
Quartile 1 (highest)	1.5 (0.2 to 2.9)	1.1 (0.3 to 1.9)	0.5 (−0.4 to 1.3)	0.2 (−1.7 to 2.1)	−0.2 (−1.7 to 1.4)	0.4 (−0.9 to 1.7)
Quartile 2	0.6 (−0.2 to 1.3)	0.6 (−0.009 to 1.1)	0.03 (−0.5 to 0.6)	1.2 (−0.2 to 2.6)	0.3 (−0.6 to 1.3)	0.8 (−0.4 to 2.1)
Quartile 3	0.8 (0.1 to 1.4)	0.3 (−0.08 to 0.8)	0.4 (0.04 to 0.8)	−0.2 (−1.5 to 1.1)	−0.4 (−1.2 to 0.5)	0.2 (−0.6 to 0.9)
Quartile 4 (lowest)	1 [Reference]	1 [Reference]	1 [Reference]	1 [Reference]	1 [Reference]	1 [Reference]
Age-adjusted drug overdose mortality rate, deaths per 100 000 population	0.03 (−0.01 to 0.07)	−0.01 (−0.04 to 0.01)	0.04 (0.009 to 0.07)	0.006 (−0.09 to 0.10)	−0.02 (−0.1 to 0.05)	0.03 (−0.03 to 0.09)
Unemployment rate, %	−0.4 (−0.7 to 0.006)	−0.3 (−0.5 to −0.09)	−0.05 (−0.3 to 0.1)	0.08 (−0.2 to 0.4)	−0.02 (−0.3 to 0.3)	0.1 (−0.1 to 0.3)
Insured adults aged 18-64 y, %	0.10 (0.03 to 0.2)	0.06 (0.008 to 0.1)	0.04 (−0.002 to 0.08)	0.08 (−0.003 to 0.2)	0.0009 (−0.07 to 0.07)	0.08 (0.02 to 0.1)
Median household income, logarithms	−1.5 (−4.1 to 1.1)	−0.3 (−1.9 to 1.3)	−1.2 (−2.6 to 0.1)	0.8 (−2.1 to 3.7)	2.0 (−0.6 to 4.5)	−1.1 (−4.1 to 1.9)
Population, logarithms	0.2 (−0.2 to 0.7)	0.3 (0.01 to 0.5)	−0.03 (−0.4 to 0.3)	0.5 (−0.01 to 1.1)	0.5 (−0.02 to 1.0)	0.06 (−0.3 to 0.5)
Dependent variable, mean (SD)	4.07 (6.14)	1.99 (4.27)	2.08 (3.61)	2.33 (8.77)	0.61 (6.06)	1.73 (6.60)

Abbreviations: APN, advanced practice nurse; PA, physician assistant.

^a^ Unless indicated otherwise, data are presented as the point estimate (95% CI) from a separate multivariable linear regression of the raw 12-month change in waiver capacity (between March 31, 2021, and March 31, 2022). The SEs were clusters in states.

## Discussion

This cross-sectional study found that waiver growth during the first year after the federal education exemption was modest and concentrated among urban counties and counties with high baseline levels of clinicians with waivers. Although most growth occurred in urban areas, APN and PA waivers accounted for more than 70% of rural growth. Our results reinforce emerging evidence that APNs and PAs play an important role in buprenorphine treatment in rural areas.^5,6^

Inability to examine effects of the exemption on waiver-seeking behavior and prescribing practices is a limitation of this study. Future qualitative research should explore why the removal of educational requirements did not significantly change clinicians’ waiver-seeking behavior and other barriers such as stigma, insufficient institutional and staff support, and limited access to counselors. Overall, our findings suggest that removing educational barriers is insufficient to address low waiver uptake among US clinicians in certain areas such as counties with preexisting low levels of uptake or rural counties.

## Notes

Supplement. **eMethods 1.** Mapping Clinicians’ Zip Codes to Their Corresponding County Codes **eMethods 2.** County-Level Rurality **eMethods 3.** Additional Details of Variables
eReferences
