# Supplementary material for: Analysis of US County Characteristics and Clinicians With Waivers to Prescribe Buprenorphine After Changes in Federal Education Requirements
Source: JAMA Netw Open. 2022 Oct 21;5(10):e2237912. doi: 10.1001/jamanetworkopen.2022.37912 (PMC9587475; doi:10.1001/jamanetworkopen.2022.37912)
Supplement: Supplement. — eMethods 1. Mapping Clinicians’ Zip Codes to Their Corresponding County Codes eMethods 2. County-Level Rurality eMethods 3. Additional Details of Variables eReferences [file jamanetwopen-e2237912-s001.pdf]

Supplementary Online Content

Nguyen T, Andraka-Christou B, Arnaudo C, Bradford WD, Simon K, Spetz J. Analysis of US county characteristics and clinicians with waivers to prescribe buprenorphine after changes in federal education requirements. *JAMA Netw Open*. 2022;5(10):e2237912. doi:10.1001/jamanetworkopen.2022.37912

eMethods 1. Mapping Clinicians' Zip Codes to Their Corresponding County Codes
eMethods 2. County-Level Rurality
eMethods 3. Additional Details of Variables
eReferences

This supplementary material has been provided by the authors to give readers additional information about their work.

© 2022 Nguyen T et al. *JAMA Network Open*.

eMethod 1. Mapping Clinicians' Zip Codes to Their Corresponding County Codes

The Controlled Substances Act Registration Information Database provides waivered clinicians' practice address, including ZIP codes. Similar to prior work (Boland et al. 2017; Nguyen et al. 2022), we mapped clinicians' ZIP codes to their corresponding Federal Information Processing Standard (FIPS) county codes using the ZIP-FIPS crosswalk file in the R package "non-census" (Ramey 2016).

eMethod 2. County-Level Rurality

We used the National Center for Health Statistics Urban-Rural Classification (NCHS) Scheme to define rural counties vs. urban counties (Centers for Disease Control and Prevention National Center for Health Statistics 2022). Specifically, we used NCHS's most rural category, nonmetropolitan "noncore" counties, to define rural counties in this study. The urban counties included metropolitan statistical areas and the micropolitan statistical areas. NCHS assigned 1,335 counties to the noncore category, 641 counties to the micropolitan category, and 1,167 counties to the metropolitan category.

eMethod 3. Additional Details of Variables

We controlled for county-level age-adjusted drug overdose mortality rate and socioeconomic characteristics (including unemployment rate, adult insurance rate, median household income, and population) to increase the precision of estimates in this study. As data for many control variables in 2020-2021 were not available, we used data of 2019 for these variables.

The county-level age-adjusted drug overdose mortality rate (drug overdose deaths per 100,000 population) was obtained from National Center for Health Statistics databases. The median

© 2022 Nguyen T et al. *JAMA Network Open*.

household income, non-white population, and population were from the County Health Rankings Database. The unemployment rate and number of active MDs were obtained from the Area Health Resources Files. The adult insurance rate was from the Small Area Health Insurance Estimates Program.

eReferences

Boland, Mary Regina, Pradipta Parhi, Pierre Gentine, and Nicholas P Tatonetti. 2017. "Climate Classification Is an Important Factor in Assessing Quality-of-Care across Hospitals." *Scientific Reports* 7(1): 4948.

Centers for Disease Control and Prevention National Center for Health Statistics. 2022. *NCHS Urban-Rural Classification Scheme for Counties*. https://www.cdc.gov/nchs/data_access/urban_rural.htm (May 1, 2022).

Nguyen, Thuy, Barbara Andraka-Christou, W David Bradford, and Kosali Simon. 2022. "Opting into the Public List of DATA-Waivered Practitioners: Variations by Specialty, Treatment Capacity, and Practitioner Characteristics." *Journal of addiction medicine* 16(3): e197–202.

Ramey, J A. 2016. "U.S. Census Regional and Demographic Data." https://cran.r-project.org/web/packages/noncensus/noncensus.pdf.

© 2022 Nguyen T et al. *JAMA Network Open*.
